# Supplementary material for: The functional neurobiology of negative affective traits across regions, networks, signatures, and a machine learning multiverse
Source: bioRxiv. 2025 May 20:2025.05.15.653674. Preprint. [Version 1] doi: 10.1101/2025.05.15.653674 (PMC12139946; doi:10.1101/2025.05.15.653674)
Supplement: Supplement 1 [file media-1.pdf]

The functional neurobiology of negative affective traits across regions, networks, signatures, and a machine learning multiverse

-Supplements-

Content

MRI data acquisition and preprocessing 2
Details on Machine Larning Algorithms 2
Method for within-person prediction 3
Supplemental tables and figures 3
Table S1 4
Table S2 5
Table S3 6
Table S4 8
Figure S1 10

MRI data acquisition and preprocessing

Functional BOLD image acquisition parameters for the facial-expression tasks were: field-of-view (FOV) = 200×200mm, matrix size = 64×64, time-to-repetition (TR) = 2000ms, time-to-echo (TE) = 29ms, and flip angle (FA) = 90°. Thirty-four slices per volume were collected along an inferior-to-superior encoding direction, with each volume having a 3mm thickness and no gap. A total of 195 and 273 BOLD signal volumes were collected throughout the facial-expression tasks using PFA and NIM-STIM images, respectively. Functional BOLD image acquisition parameters for the IAPS task were: FOV = 205×205mm, matrix size = 64×64, TR = 2000ms, TE = 28ms, and FA = 90°. The entire task duration was 11 min and 16 sec (15 'Look neutral' trials; 15 'Look negative' trials; 15 'Decrease negative' trials). Images were presented such that no more than 2 identical trial types ("Look negative" or "Decrease negative") were consecutive, and no more than 4 unpleasant images were consecutive. Thirtynine slices per volume were collected along an inferior-to-superior encoding direction, with each volume having a 3mm thickness and no gap (338 task volumes in total). A 6-sec countdown preceded task onset. The 3 volumes of this countdown were not modeled in the design matrix, nor were remaining 3 volumes collected after the offset of the final rest period (344 functional run volumes in total). To co-register BOLD signal volumes, T1-weighted magnetization prepared rapid gradient echo (MPRAGE) images were acquired over 7 min and 17 sec by these parameters: FOV = 256×208mm, matrix size = 256×208, TR = 2100ms, inversion time (TI) = 1100ms, TE = 3.31ms, and FA = 8° (192 slices, 1mm thickness, no gap).

All fMRI data for the facial-expression and IAPS tasks were preprocessed with statistical parametric mapping software (SPM12; http://www.fil.ion.ucl.ac.uk/spm). For spatial preprocessing, T1-weighted MPRAGE images were classified into 6 tissue types. Biasedcorrected and deformation field maps were then computed. Functional images were realigned to the first image of the series by 6-parameter rigid-body transformation, using the re-slice step to match the first image on a voxel-by-voxel basis. Before realignment, slice-timing correction was applied to IAPS fMRI task data to account for acquisition time variation in this eventrelated design. Realigned images were co-registered to each participant's skull-stripped and biased-corrected MPRAGE image. Co-registered images were then normalized to Montreal Neurological Institute (MNI) space. Normalized images were then smoothed by a 6mm fullwidth-at-half-maximum (FWHM) Gaussian kernel.

Details on Machine Larning Algorithms

Model performance in the training sample was estimated using 2x5x5 nested cross-validation the inner folds being used for hyperparameter optimization, and the outer folds for independent testing using the chosen optimal hyperparameter (PLS/PCR: number of components between one and the sample size of the innermost training fold optimized using Bayesian parameter optimization, SVM: Slack parameter C optimized using grid search, RF: mtry with grid search between one and total number of variables after dimensionality reduction using PCA with the number of components equal to the sample size of the innermost training fold). The procedure was repeated two times for more stability. Product-

moment correlations were used as a performance metric, as the aim was to train a pattern for scale-independent correlates of negative affectivity, which can be used across different studies with different scanning parameters or different questionnaires. The resulting 10 product-moment correlations (two repeats times five outer folds) were Fisher-transformed, averaged, and the average back-transformed for the final model estimate. The model configuration with the highest average correlation was chosen for evaluation in the hold-out sample. Before evaluation, the model was retrained on the full training data using an optimal hyperparameter chosen based on 5-fold cross-validation (corresponding to the inner folds of the nested procedure). Using this final model, neuroticism scores of the hold-out sample were correlated with model predictions, generated by taking the dot product between machine learning pattern and fMRI data. This final correlation was supplemented with p-values and Bayes factors, both one-sided.

Method for within-person prediction

Within-person prediction had been previously performed on the same dataset for the creation of the PINES signature by Chang and colleagues (2015; see main manuscript). They prepared one beta image for each person and rating to a negative picture, i.e., as ratings varied between 1 and 5, each participant is associated with five beta images. These images are openly available on neurovault: https://neurovault.org/collections/1964/. In accordance with the previous study, we stacked the images on top of each other in a single data matrix. Then, we performed 10-fold cross-validation, leaving out 10% of participants in each iteration to avoid dependencies in training and test data. Nine folds were used for training a predictive model based on the specifications of the best model we identified for individual differences, i.e., support vector regression after image-wise centering. In the remaining test fold, a correlation between actual and predicted ratings was calculated separately for each participant. This procedure was repeated until each participant was part of a test fold, providing person-specific correlations for the whole sample. We report the mean value and standard deviation for these person-wise correlations in the main paper.

Table S1

Descriptive statistics for psychological traits

Construct	*M*	*SD*	1	2	3	4	5	6	7	8	9	10	11	12	13
1. Neuroticism	75.5	21.7													
2. N1: Anxiety	13.7	5.2	0.81												
3. N2: Hostility	12.0	5.0	0.74	0.52											
4. N3: Depression	11.5	5.6	0.84	0.63	0.54										
5. N4: Self-Consciousness	13.8	4.5	0.71	0.55	0.36	0.55									
6. N5: Impulsiveness	15.4	4.6	0.59	0.34	0.39	0.35	0.25								
7. N6: Vulnerability	9.1	4	0.80	0.6	0.51	0.67	0.52	0.37							
8. N: Other-Report	16.2	9.1	0.42	0.36	0.37	0.36	0.27	0.19	0.34						
9. Negative Affect	15.8	5.3	0.62	0.53	0.45	0.62	0.38	0.30	0.48	0.29					
10. STAI	32.5	8.4	0.71	0.58	0.49	0.71	0.47	0.30	0.63	0.39	0.59				
11. BDI	4.2	4.4	0.44	0.31	0.32	0.50	0.26	0.23	0.36	0.33	0.45	0.62			
12. Positive Affect	34.5	6.5	-0.33	-0.21	-0.22	-0.32	-0.28	-0.12	-0.37	-0.13	-0.11	-0.37	-0.29		
13. Extraversion	115	19.5	-0.33	-0.23	-0.18	-0.31	-0.39	-0.04	-0.36	-0.11	-0.21	-0.30	-0.17	0.49	
14. Task-Ratings	2.4	0.7	0.03	0.10	-0.03	-0.01	0.12	-0.07	0.03	-0.02	0.02	-0.03	-0.10	-0.02	0.09

Note. STAI = State-Trait Anxiety Inventory (trait scores). BDI = Beck Depression Inventory. Task-Ratings = Person-wise average difference between affective task ratings between negative and neutral scenes. Sample sizes of correlations can differ due to missingness and study-related factors (e.g., many participants only performed the faces task, which does not include task-based ratings, and many participants did not contribute other reports for neuroticism)

Table S2

Theory-driven neural associations with neuroticism

	IAPS (N = 332)			FACES (N = 424)		
	r	95% CI	BF_{01}	r	95% CI	BF_{01}
			Regions			
Averaged signal						
Amygdala-L	.03	[-.08, .14]	19.4	.07	[-.03, .16]	10.5
Amygdala-R	.05	[-.06, .15]	16.4	.06	[-.04, .15]	12.6
aInsula-L	-.05	[-.15, .06]	16.1	.03	[-.06, .13]	20.4
aInsula-R	-.06	[-.17, .05]	12.7	.05	[-.05, .14]	16.6
dACC-L	-.11	[-.22, .00]	3.4	.04	[-.06, .13]	18.7
dACC-R	-.11	[-.22, -.00]	2.84	.04	[-.05, .13]	17.0
PLS Models[a]						
Amygdala	-.05	[-.24, .15]	11.5	.13	[-.06, .32]	5.3
aInsula	-.05	[-.24, .15]	11.4	.02	[-.18, .21]	12.6
dACC	.04	[-.16, .23]	11.9	.09	[-.11, .28]	8.6
Best region						
Cerebellum/ Diencephalon	-.12	[-.26, .03]	4.95	.07	[-.06, .21]	9.5
			Networks			
Averaged Signal						
Visual	-.02	[-.13, .09]	21.6	.04	[-.06, .13]	19.4
Somatomotor	-.02	[-.12, .09]	21.9	.11	[.02, .20]	1.9
dAttention	-.03	[-.13, .08]	20.5	.07	[-.03, .16]	9.5
vAttention	-.04	[-.15, .07]	17.5	.07	[-.03, .16]	9.9
Limbic	-.01	[-.12, .09]	22.2	.12	[.03, .22]	1.0
Frontoparietal	-.10	[-.20, .01]	4.7	.04	[-.05, .14]	17.0
Default-Mode	-.07	[-.18, .04]	10.1	.06	[-.04, .15]	12.5
Linear Regression						
All networks[b]	-.03	.467	>100	.12	.071	>100
Random Forest						
All networks[b]	.10	.059		-.06	.710	
			Signatures			
PINES	-.09	[-.20, 0.02]	5.5	.07	[-.03, .16]	9.5
VIFS	.04	[-.07, .15]	17.6	-.03	[-.13, .07]	21.3
Fear	.05	[-.06, .15]	16.2	.01	[-.09, .10]	25.5
Anger	.01	[-.10, .12]	22.4	.05	[-.05, .14]	16.1
Sadness	-.03	[-.13, .08]	20.6	.05	[-.05, .14]	15.4

Note. BF_{01} = Bayes factor of the null hypothesis over the alternative hypothesis. Bayes factors were calculated with the bayesFactor toolbox in matlab.

[a]The proportion of in-region voxels weights with positive signs ranged between 49-51%.

[b]For the linear multiple regression approach, the multiple correlation coefficient is reported, calculated as the square-root of adjusted R^2. Here, p-values are reported instead of confidence intervals, as confidence intervals for (unadjusted) R^2 cannot contain zero and are therefore harder to interpret in terms of statistical significance. Similarly, p-values are reported for the random forest approach, as it was preregistered to be conducted on the whole sample with out-of-bag prediction for which (to our knowledge) currently are no valid confidence intervals available. The p-value is for a one-sided test, as negative correlations between predictions and actual values are not meaningful.

Table S3

Clusters of voxels with positive weights at FDR of q = .10

Region	Volume [mm³]	MNI X	Y	Z	Z_{max}-Statistic	% covered
Basal ganglia	64	-12	-14	-4	3.652305768	100
Basal ganglia	64	-12	10	12	3.852284309	100
Basal ganglia	144	32	-32	0	3.90804411	89
Basal ganglia	160	-24	-16	8	4.104912286	100
Brainstem	224	10	-24	-34	4.04452717	79
Brainstem	224	-2	-26	-12	3.983246299	43
Brainstem	472	-4	-10	-22	4.454181836	32
Cerebellum	64	28	-56	-42	3.545403913	25
Cerebellum	64	8	-44	-42	3.73168988	100
Cerebellum	64	38	-74	-38	3.666787978	88
Cerebellum	64	-22	-60	-36	3.601441673	88
Cerebellum	128	22	-54	-36	4.052773897	75
Cerebellum	144	34	-68	-40	3.96928996	67
Cerebellum	144	12	-48	-16	4.026818128	56
Cerebellum	208	18	-70	-14	4.175139382	62
Cerebellum	416	12	-66	-38	3.895829671	54
Cerebellum	776	-12	-68	-38	4.334853742	35
Cortex Default ModeA	128	-22	20	44	3.605726765	50
Cortex Default ModeA	144	-8	62	8	3.953326055	100
Cortex Default ModeB	64	-58	-46	-2	3.681519856	50
Cortex Default ModeB	128	-42	24	-14	3.650804356	75
Cortex Default ModeB	208	16	48	26	4.272855498	35
Cortex Default ModeC	64	34	-26	-24	3.566796028	63
Cortex Default ModeC	200	-38	-26	-18	3.786977859	52
Cortex Dorsal AttentionA	256	28	-60	30	4.303589365	41
Cortex Dorsal AttentionA	392	24	-78	20	3.996921822	33
Cortex Dorsal AttentionA	432	40	-42	66	4.388786267	13
Cortex Dorsal AttentionB	64	12	-54	60	3.927093939	100
Cortex Dorsal AttentionB	64	-42	-52	62	3.646879984	75
Cortex Dorsal AttentionB	216	-38	-4	32	4.343334203	7
Cortex Dorsal AttentionB	296	-32	-54	68	4.411285513	84
Cortex Fronto ParietalA	128	28	14	28	4.304903366	25
Cortex Fronto ParietalB	128	-58	-50	-16	3.943971404	100
Cortex Fronto ParietalB	288	64	-20	-18	3.843402842	75
Cortex Fronto ParietalC	256	0	-74	44	3.597457891	50
Cortex Fronto ParietalC	432	-2	-72	52	4.254097528	43
Cortex Limbic	64	18	-4	-42	3.529895563	25
Cortex Limbic	128	60	-16	-34	3.779750831	63
Cortex Limbic	160	28	0	-38	3.806306957	55
Cortex Limbic	176	44	20	-20	4.221152529	73
Cortex Limbic	176	16	52	-18	3.893277031	68

Cortex Limbic	288	54	-12	-36	4.256764093	89
Cortex Limbic	320	54	8	-32	3.992710966	98
Cortex Limbic	424	22	-8	-44	4.367441661	28
Cortex Limbic	520	-28	0	-40	4.086828182	66
Cortex Limbic	736	36	20	-38	5.03981465	97
Cortex SomatomotorB	600	40	-18	2	3.833716071	28
Cortex Temporal Parietal	256	-48	6	-6	4.158587209	41
Cortex Ventral AttentionA	192	42	-4	-4	4.009962572	79
Cortex Ventral AttentionA	320	38	-14	-10	4.447322313	25
Cortex Ventral AttentionB	64	-42	46	28	3.958137593	100
Cortex Ventral AttentionB	224	48	38	14	4.141441415	21
Cortex Ventral AttentionB	272	2	32	18	4.040992933	82
Cortex Visual Central	64	40	-68	-20	3.586643375	50
Cortex Visual Central	128	-16	-88	12	3.842890959	6
Cortex Visual Central	336	2	-84	22	3.910583629	38
Cortex Visual Peripheral	64	-8	-70	-2	3.536887002	100
Cortex Visual Peripheral	64	-22	-74	2	3.534790704	50
Cortex Visual Peripheral	64	-24	-86	36	3.593700695	75
Cortex Visual Peripheral	384	8	-62	6	4.277608272	83
Cortex Visual Peripheral	552	26	-64	6	4.035739112	49
Diencephalon	64	18	-24	-4	3.57039058	100
No description	64	68	-48	4	3.524026159	0
No description	64	24	22	10	3.547524833	0
No description	64	28	-58	12	3.588740176	0
No description	64	28	-16	52	3.526708569	0
No description	64	-22	-76	58	4.050048247	0
No description	128	44	-40	28	4.08176954	0

Note. The "% covered" column indicates the percent of voxels in a cluster cover by a specific region label. Automated labelling was performed with the canlab toolbox using the region() and table() functions.

Table S4

Clusters of voxels with negative weights at FDR of q = .10

Region	Volume [mm³]	MNI X	MNI Y	MNI Z	Z_{max}-Statistic	% covered
Basal ganglia	64	6	0	-10	-3.54718	75
Basal ganglia	64	2	12	16	-3.7611	50
Basal ganglia	88	0	14	14	-3.55865	9
Basal ganglia	144	-24	12	-4	-3.80977	72
Basal ganglia	264	-8	0	18	-4.20653	100
Basal ganglia	384	-18	-10	28	-4.18908	79
Basal ganglia	560	14	28	0	-4.40146	36
Basal ganglia	784	18	-32	10	-4.48621	44
Cerebellum	288	4	-56	-48	-4.21364	100
Cerebellum	336	0	-64	-6	-4.4739	57
Cortex Default ModeA	64	-40	-68	34	-3.51907	38
Cortex Default ModeA	64	-2	-50	36	-3.61961	50
Cortex Default ModeA	160	-8	20	-12	-3.65508	50
Cortex Default ModeA	176	-4	52	-8	-3.89427	68
Cortex Default ModeA	192	10	-48	44	-4.02499	46
Cortex Default ModeB	64	-14	40	44	-3.53953	50
Cortex Default ModeB	64	-54	-6	52	-3.60081	88
Cortex Default ModeB	96	-56	4	-36	-3.61481	100
Cortex Default ModeB	192	-32	0	-32	-4.00529	21
Cortex Default ModeB	208	-48	28	0	-4.05867	81
Cortex Default ModeB	264	-52	-10	44	-4.08027	48
Cortex Dorsal AttentionA	400	52	-58	8	-3.94604	88
Cortex Dorsal AttentionA	824	50	-68	-2	-4.44292	24
Cortex Dorsal AttentionB	64	-44	-32	40	-3.60794	75
Cortex Dorsal AttentionB	96	34	-30	52	-3.79994	58
Cortex Dorsal AttentionB	208	-38	2	56	-3.77187	88
Cortex Dorsal AttentionB	392	14	-44	70	-4.2037	49
Cortex Dorsal AttentionB	808	38	0	54	-4.85951	63
Cortex Fronto ParietalA	128	32	-48	52	-3.75713	69
Cortex Fronto ParietalA	288	42	16	16	-4.27468	8
Cortex Fronto ParietalA	336	38	-2	40	-4.4202	31
Cortex Fronto ParietalA	928	48	20	34	-4.48172	31
Cortex Fronto ParietalB	128	-6	20	40	-3.84357	88

Cortex Fronto ParietalB	176	8	44	36	-4.37012	9
Cortex Fronto ParietalB	176	28	26	48	-4.09105	82
Cortex Fronto ParietalB	200	58	-56	46	-3.92567	4
Cortex Fronto ParietalB	224	14	38	-18	-4.16718	18
Cortex Limbic	144	-2	38	-30	-3.92036	44
Cortex Limbic	160	16	14	-16	-3.81417	30
Cortex Limbic	176	36	12	-28	-4.45452	77
Cortex SomatomotorA	64	-62	-10	22	-3.55048	75
Cortex SomatomotorA	128	-54	-28	48	-3.64743	50
Cortex SomatomotorA	904	-30	-20	60	-4.93739	27
Cortex SomatomotorB	192	46	-26	22	-3.9968	71
Cortex Temporal Parietal	128	-60	-16	-4	-3.85981	31
Cortex Ventral AttentionA	64	34	14	-18	-3.68263	75
Cortex Ventral AttentionA	112	-54	-36	48	-3.72868	57
Cortex Ventral AttentionA	128	12	-12	44	-3.78576	44
Cortex Ventral AttentionA	192	66	-20	40	-3.74956	8
Cortex Ventral AttentionA	192	-2	0	56	-3.7675	46
Cortex Ventral AttentionB	64	-20	44	-18	-3.56508	100
Cortex Ventral AttentionB	64	36	22	8	-3.59033	100
Cortex Ventral AttentionB	616	4	36	30	-4.72523	60
Cortex Visual Central	192	42	-80	-4	-3.94547	88
Cortex Visual Central	336	0	-84	-12	-3.86713	48
Cortex Visual Central	344	-42	-76	2	-4.60834	40
Cortex Visual Peripheral	344	6	-40	-2	-4.07015	37
Diencephalon	176	-2	-4	6	-3.5862	36
Diencephalon	272	0	-26	8	-4.42874	18
Hippocampus	96	12	-38	4	-3.57135	17
No description	64	-44	-46	-4	-3.605	0
No description	64	16	46	-4	-3.64018	0
No description	64	-30	16	32	-3.61792	0
No description	128	-20	-44	16	-3.79467	0
No description	224	-26	-46	12	-4.45522	0

Note. The “% covered” column indicates the percent of voxels in a cluster cover by a specific region label. Automated labelling was performed with the canlab toolbox using the region() and table() functions.

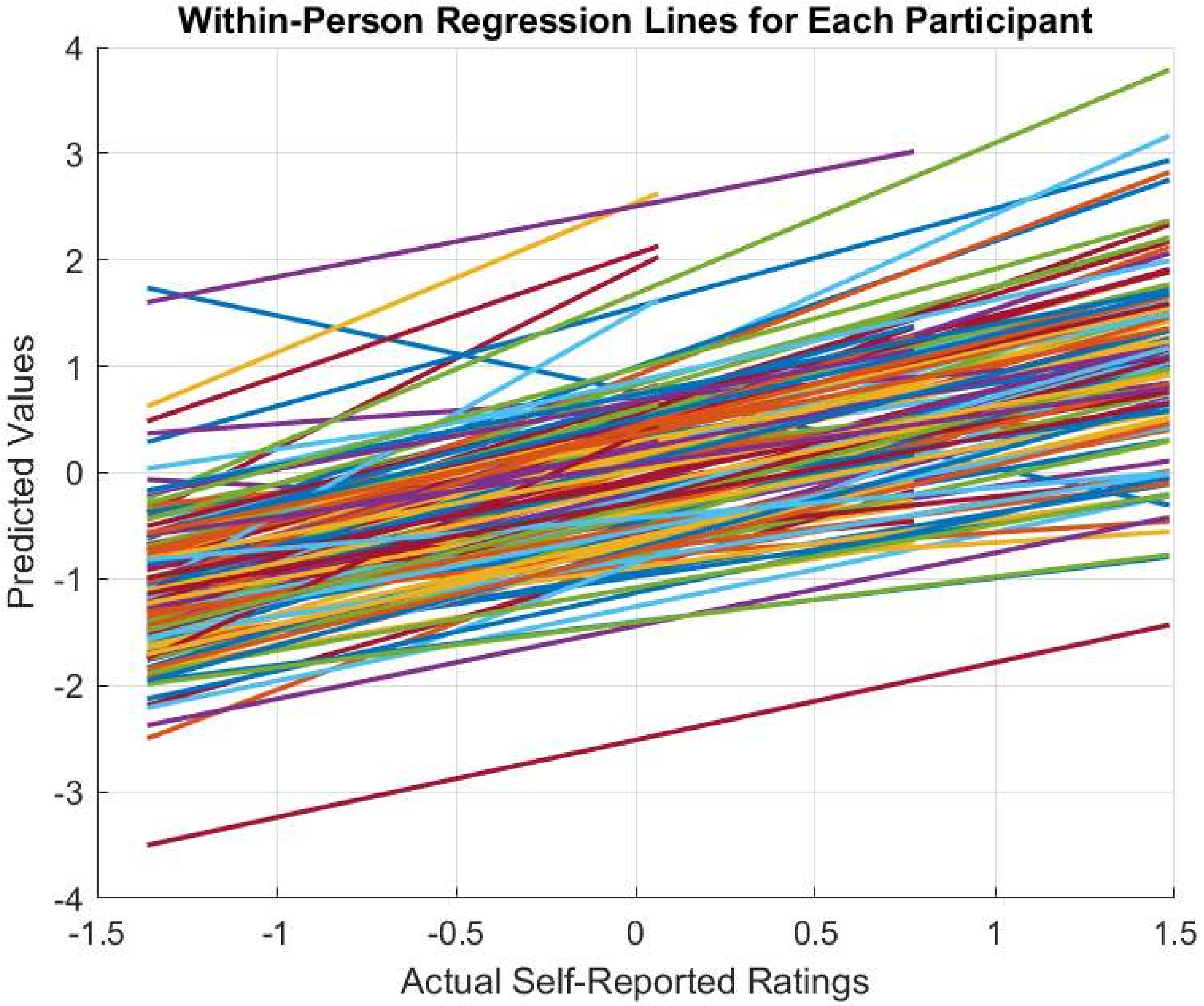

Figure S1.
Within-person regression lines for the association between actual state-based self-report rating of negative affect and predicted values from a support vector regression.
